# Primary Diffuse Large B‐Cell Lymphoma of the Seminal Vesicles: A Rare Case With Diagnostic and Therapeutic Implications

**DOI:** 10.1002/ccr3.70489

**Published:** 2025-05-27

**Authors:** Mohammad Soleimani, Mostafa Farajpour, Behrang Kazeminejad

**Affiliations:** ^1^ Department of Urology Shahid Beheshti University of Medical Science, Modarres Hospital Tehran Iran; ^2^ Department of Pathology Shahid Beheshti University of Medical Science, Modarres Hospital Tehran Iran

**Keywords:** DLBCL, hydroureteronephrosis, lymphoma, pelvic mass, R‐CHOP, seminal vesicle

## Abstract

Primary diffuse large B‐cell lymphoma of the seminal vesicles is rare but treatable. Multimodal imaging and biopsy confirmed the diagnosis in a 68‐year‐old male. Eight cycles of R‐CHOP chemotherapy achieved a favorable outcome, emphasizing early intervention in atypical pelvic masses.

## 1 Introduction

Primary tumors of the seminal vesicles are uncommon, with most cases representing secondary involvement from adjacent organs (e.g., prostate and bladder) or distant metastases. The incidence of primary seminal vesicle tumors is extremely low, estimated at less than 0.1% of all urological malignancies [1]. Among these, primary seminal vesicle lymphomas, particularly diffuse large B‐cell lymphoma (DLBCL)—the most common subtype of non‐Hodgkin lymphoma—are exceptionally rare, with fewer than 10 cases reported in the literature [2, 3, 4]. Lymphomas are broadly classified into Hodgkin and non‐Hodgkin lymphomas, with DLBCL accounting for approximately 30%–40% of non‐Hodgkin cases [5]. The most frequent seminal vesicle tumors include adenocarcinomas and secondary metastases, while lymphomas remain a diagnostic rarity [6]. Unlike typical extranodal lymphoma sites (e.g., gastrointestinal tract and skin), the seminal vesicles present unique diagnostic difficulties due to their rarity and nonspecific symptoms, often mimicking urological conditions. This report describes a rare case of primary DLBCL of the seminal vesicles, aiming to enhance clinician awareness, outline diagnostic challenges, and discuss therapeutic strategies based on a 68‐year‐old male patient's presentation and management.

## 2 Case Presentation

### 2.1 Clinical History

A 68‐year‐old male presented with a 4‐week history of intermittent, dull flank pain that progressively worsened. He had no prior hematuria, renal, or prostate issues; last year's abdominopelvic sonography was normal. Physical examination revealed flank discomfort on palpation. Digital rectal examination (DRE) identified a palpable mass in the right seminal vesicle, raising suspicion of an abscess or malignancy.

### 2.2 Diagnostic Assessment

**FIGURE 1:**
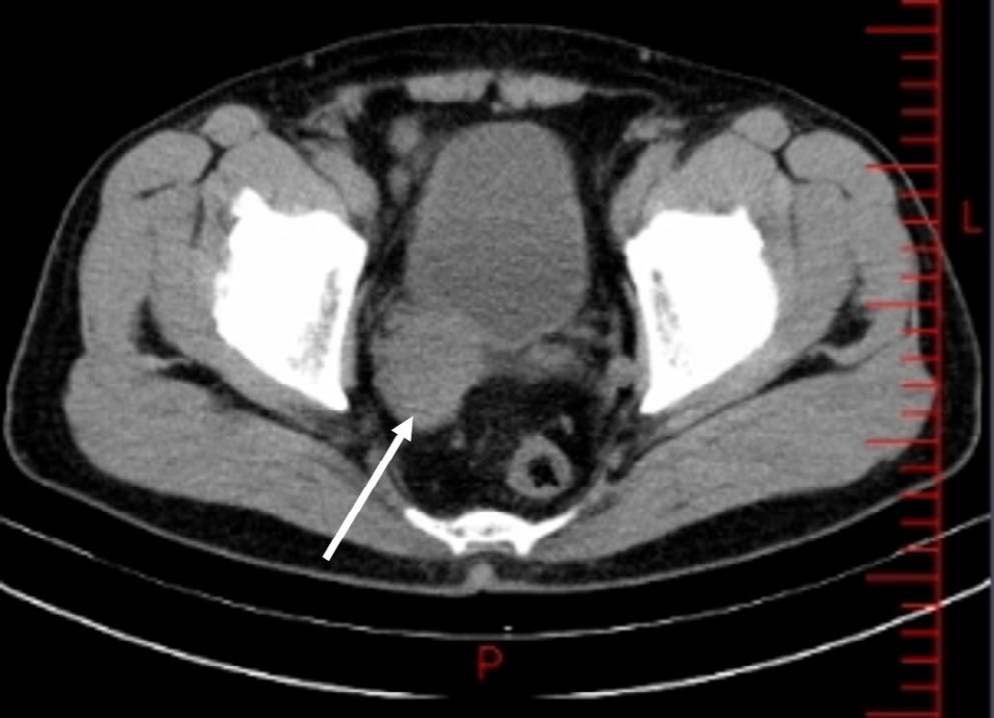
Axial CT scan showing a 44 × 35 mm mass in the right seminal vesicle causing ureteral compression.

**FIGURE 2:**
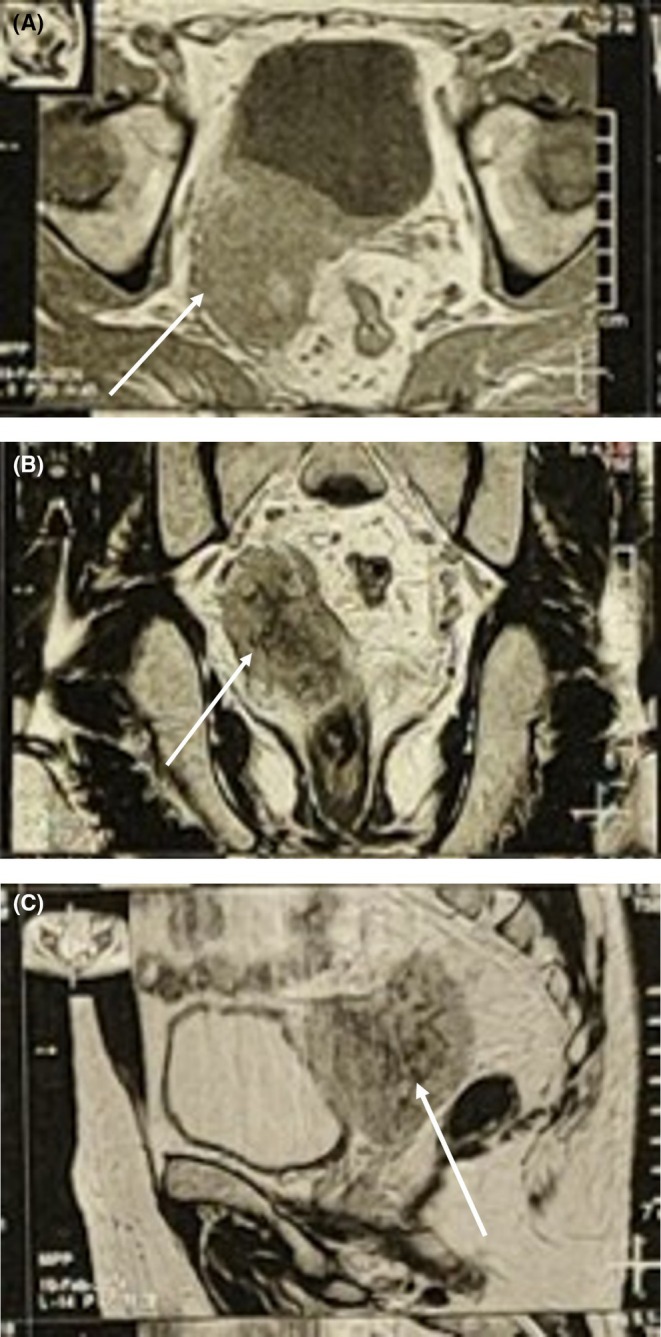
Biparametric MRI of the right seminal vesicle mass. (A) Axial T1‐weighted image showing a hyperintense mass with adjacent compression. (B) coronal T2‐weighted image demonstrating the mass with ureteral compression effects. (C) Sagittal T2‐weighted image highlighting the extent of the mass and its impact on surrounding structures.

Laboratory results showed elevated creatinine at 2.5 mg/dL, suggesting renal impairment possibly due to ureteral obstruction. Imaging studies included:

– Abdominopelvic sonography: right hydroureteronephrosis.
– CT Scan (non‐contrast): a 44 × 35 mm soft‐tissue mass in the right seminal vesicle, compressing the ureter, with no calcification (Figure 1).
– Bi‐parametric MRI: a hyperintense T2‐weighted, hypointense T1‐weighted mass in the right seminal vesicle, consistent with malignancy (Figure 2).
– PET‐CT: hypermetabolic mass involving the seminal vesicle, prostate, and bladder wall; hypermetabolic lymph nodes in bilateral iliac, para‐aortic, and mediastinal regions; and two foci in the gastric body.
– Chest CT: no evidence of metastasis.

**FIGURE 3:**
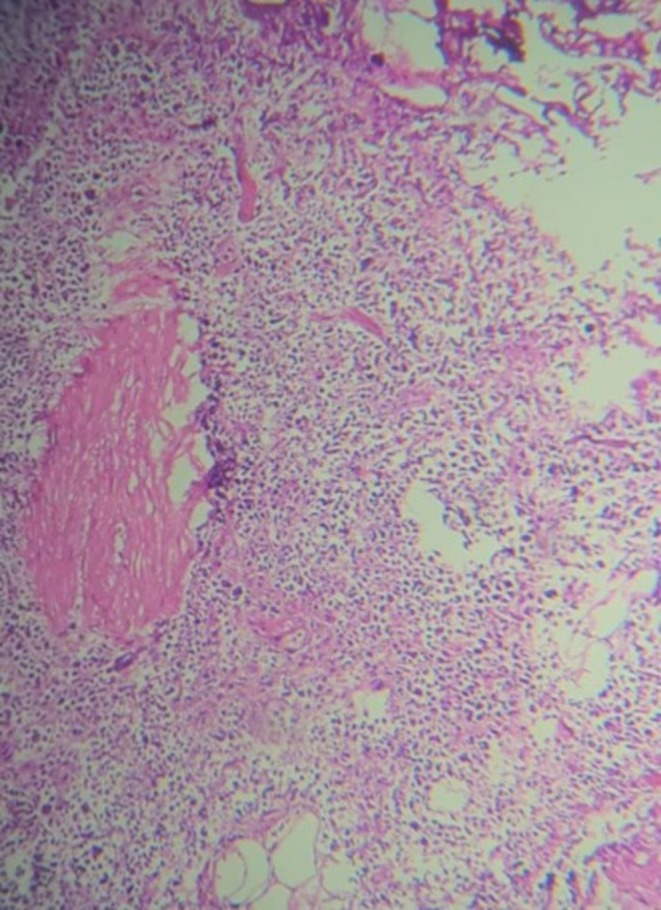
Immunohistochemical staining for CD20 (×400) showing diffuse membranous positivity in tumor cells.

**FIGURE 4:**
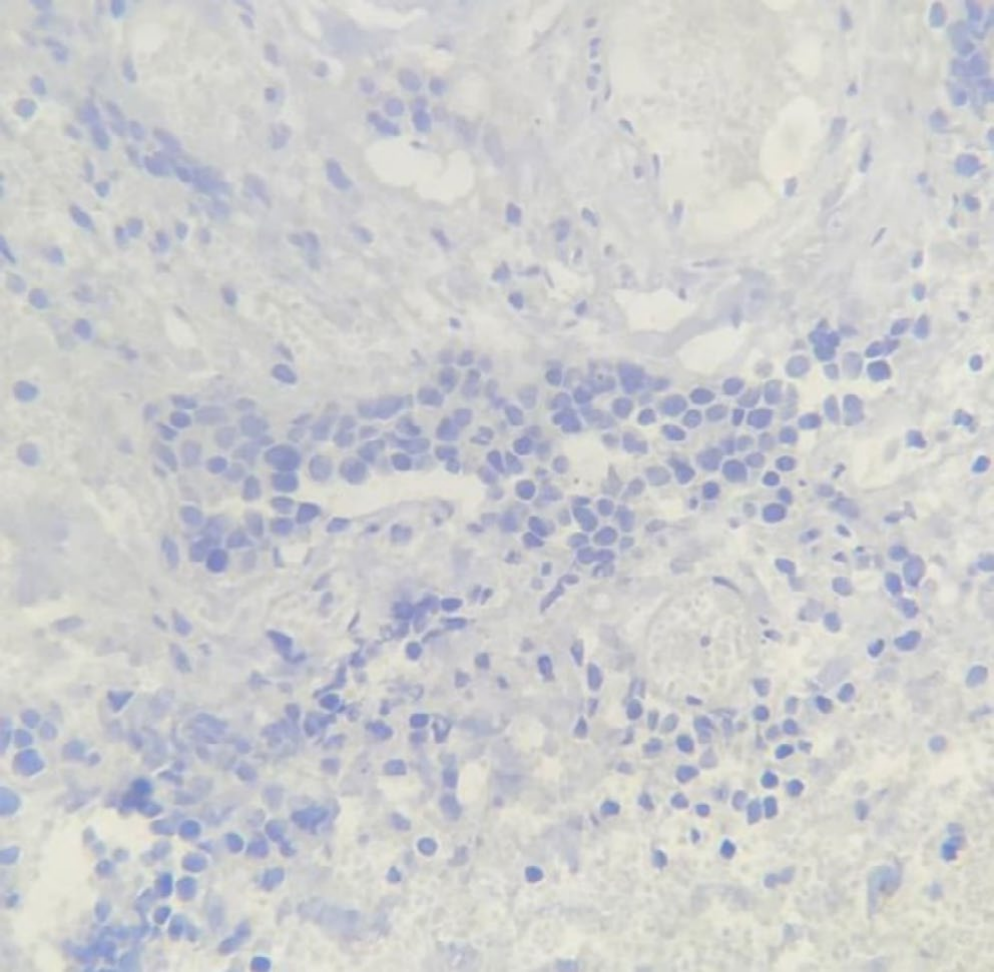
Immunohistochemical staining for PSA (×400) showing negativity in tumor cells.

Histopathology and immunohistochemistry (IHC) from a biopsy revealed large, atypical tumor cells with vesicular nuclei and prominent nucleoli. No necrosis was observed in the biopsy sample, and there was no microscopic evidence of involvement of adjacent structures in the sampled tissue. IHC showed: CD20+ (diffusely positive), CD79a+ (positive), BCL2+ (80%), BCL6+ (40%), CD10− (negative), MUM1+ (80%), MYC+ (scatteredly positive, 10%), CD30− (negative), CD3− (negative), ALK1− (negative), CK− (negative), PSA− (negative), and Ki67 proliferation activity index up to 70%, confirming DLBCL (Figures 3 and 4). The negativity of CD10 and positivity of MUM1 suggest a non‐germinal center B‐cell (non‐GCB) subtype, which may have prognostic implications [7]. The negativity of PSA and CK rules out a prostatic or epithelial origin, supporting the primary seminal vesicle origin of the tumor. The negativity of CD3, CD30, and ALK1 further excludes other hematopoietic malignancies such as T‐cell lymphomas or ALK‐positive large cell lymphoma. However, PET‐CT imaging indicated involvement of the prostate, bladder wall, regional lymph nodes, and distant sites (gastric foci), suggesting local and systemic spread, which could not be confirmed histologically due to the limitations of biopsy sampling.

### 2.3 Treatment

The patient underwent eight cycles of R‐CHOP (rituximab 375 mg/m^2^, cyclophosphamide 750 mg/m^2^, doxorubicin 50 mg/m^2^, vincristine 1.4 mg/m^2^, prednisone 100 mg/day for 5 days) over 5 months. Filgrastim was used to manage neutropenia. No significant dose adjustments were required. Written informed consent was obtained from the patient for publication of this case report.

### 2.4 Differential Diagnosis

Initial considerations included urinary tract malignancy, benign prostatic hyperplasia, secondary seminal vesicle tumors, or abscess, prompted by hydroureteronephrosis and DRE findings. DLBCL was confirmed post‐biopsy, ruling out alternatives such as sarcoma or benign vesicular tumors due to IHC and imaging characteristics. The negativity of PSA and CK further excluded a prostatic or epithelial origin, while the negativity of CD3, CD30, and ALK1 ruled out other hematopoietic malignancies such as T‐cell lymphomas or ALK‐positive large cell lymphoma.

### 2.5 Conclusion and Results (Outcome and Follow‐Up)

Follow‐up PET‐CT at 3 and 6 months showed a marked response: the pelvic mass reduced in size and metabolic activity, and hypermetabolic lymph nodes resolved. A persistent hypermetabolic focus in the right prostate lobe prompted a TRUS‐guided biopsy, which was negative for malignancy. The gastric foci were not pursued further due to clinical improvement and lack of symptoms.

## 3 Discussion

Primary DLBCL of the seminal vesicles is an exceedingly rare entity, with fewer than 10 cases reported in the literature [1, 2, 3, 4]. This rarity poses significant diagnostic challenges, as the condition often mimics more common urological conditions such as abscesses, benign prostatic hyperplasia, or secondary tumors. In our case, the patient presented with nonspecific symptoms, including flank pain and hydroureteronephrosis, which initially suggested a urinary tract issue.

Clinically, the presentation of seminal vesicle DLBCL can vary widely, ranging from asymptomatic masses to obstructive symptoms, as seen in our patient with ureteral compression. Digital rectal examination (DRE) was pivotal in identifying a palpable mass, highlighting the importance of a thorough physical examination in such cases.

Imaging played a crucial role in the diagnostic process. Abdominopelvic sonography revealed hydroureteronephrosis, while CT and MRI confirmed a 44 × 35 mm mass in the right seminal vesicle with hyperintense T2‐weighted and hypointense T1‐weighted signals, consistent with malignancy. PET‐CT further delineated the extent of disease, showing involvement of the prostate, bladder wall, and hypermetabolic lymph nodes in multiple regions, as well as gastric foci. These findings underscore the value of multimodal imaging in evaluating rare extranodal lymphomas.

Histopathologically, the biopsy revealed large, atypical tumor cells with vesicular nuclei and prominent nucleoli. No necrosis was observed in the biopsy sample, suggesting that the tumor may not have exhibited rapid growth leading to necrosis. Additionally, there was no microscopic evidence of involvement of adjacent structures in the sampled tissue; however, this finding is limited by the nature of the biopsy, as imaging (PET‐CT) indicated involvement of the prostate, bladder wall, regional lymph nodes, and distant sites (gastric foci), suggesting local and systemic spread. Immunohistochemistry (IHC) confirmed DLBCL with diffuse CD20 and CD79a positivity, BCL2 (80%), BCL6 (40%), MUM1 (80%), and a high Ki67 index (up to 70%). The negativity of CD10 and positivity of MUM1 suggest a non‐germinal center B‐cell (Non‐GCB) subtype, which may have prognostic implications [7]. The MYC expression (10%) indicates a relatively low level of MYC protein, which is associated with a less aggressive phenotype in DLBCL, although the high Ki67 index (70%) suggests significant proliferative activity. The negativity of PSA and CK rules out a prostatic or epithelial origin, supporting the primary seminal vesicle origin of the tumor. The negativity of CD3, CD30, and ALK1 further excludes other hematopoietic malignancies such as T‐cell lymphomas or ALK‐positive large cell lymphoma. However, the absence of additional markers such as cyclin D1, CD5, and EBV/EBER, as well as the lack of intensity grading for some IHC reactions (e.g., CD20 and CD79a), limits the diagnostic precision, as noted in the limitations.

Differential diagnosis initially included urinary tract malignancy, benign prostatic hyperplasia, secondary seminal vesicle tumors, and abscesses, prompted by hydroureteronephrosis and DRE findings. DLBCL was confirmed post‐biopsy, ruling out alternatives like sarcoma or benign vesicular tumors due to IHC and imaging characteristics. The negativity of PSA and CK further excluded a prostatic or epithelial origin, while the negativity of CD3, CD30, and ALK1 ruled out other hematopoietic malignancies such as T‐cell lymphomas or ALK‐positive large cell lymphoma.

Therapeutically, the patient received eight cycles of R‐CHOP chemotherapy, which resulted in a marked response, with significant reduction in tumor size and metabolic activity, as well as resolution of hypermetabolic lymph nodes. This outcome aligns with previous reports of seminal vesicle DLBCL, where R‐CHOP has shown efficacy [5, 6]. However, a persistent prostatic lesion necessitated further biopsy, which was negative for malignancy, highlighting the need for vigilant follow‐up. Compared to prior reports, such as Zhu et al. [2], who noted irreversible renal failure, our patient's creatinine elevation was reversible, suggesting obstruction rather than parenchymal damage.

Limitations of this study include the short follow‐up period, lack of genetic subtyping (e.g., germinal center vs. activated B‐cell DLBCL), and the absence of additional IHC markers (e.g., cyclin D1, CD5, and EBV/EBER) to further confirm the diagnosis. Additionally, the biopsy‐based histopathological assessment could not fully evaluate the lesion's microscopic architecture, its relationship with adjacent structures, or the presence of necrosis beyond the sampled tissue. While imaging suggested local and systemic spread, this could not be confirmed histologically due to the limitations of biopsy sampling, and further evaluation (e.g., surgical resection) would be required for a definitive assessment. A multidisciplinary approach involving urology, oncology, and pathology was critical to success, emphasizing its necessity in managing rare extranodal lymphomas.

## 4 Conclusion

This case highlights primary DLBCL of the seminal vesicles as a rare but critical differential in pelvic masses. Multimodal diagnostics, including imaging and histopathology, combined with early R‐CHOP initiation, were pivotal to achieving a favorable outcome. Clinicians should remain vigilant for lymphoma in atypical urological presentations.

## Author Contributions

**Mohammad Soleimani:** conceptualization, methodology. **Mostafa Farajpour:** conceptualization, data curation, investigation, writing – original draft. **Behrang Kazeminejad:** data curation, investigation, writing – review and editing.

## Consent

Written informed consent was obtained from the patient for the publication of this case report and any accompanying images.

## Conflicts of Interest

The authors declare no conflicts of interest.

## Data Availability

The data that support the findings of this study are not publicly available due to privacy and ethical restrictions, as they contain sensitive patient information.
